# 2947. Improving Access to Allergy Specialty Care in Houston through a Collaborative Penicillin Allergy Stewardship Program

**DOI:** 10.1093/ofid/ofad500.186

**Published:** 2023-11-27

**Authors:** Margaret G Taylor, Sara Anvari

**Affiliations:** Baylor College of Medicine, Houston, TX; Baylor College of Medicine / Texas Children's Hospital, Houston, Texas

## Abstract

**Background:**

Approximately 5% of children carry a penicillin (PCN) allergy label, but less than 10% of reported reactions are IgE-mediated. Guidelines recommend formal evaluation of children with unconfirmed PCN allergy labels, but this evaluation occurs infrequently. We piloted a novel pediatric PCN allergy clinic in the infectious disease and allergy & immunology clinics, utilizing telemedicine services to provide prompt initial PCN allergy evaluation for children in the community with unconfirmed PCN allergy labels.

**Methods:**

A collaborative PCN allergy clinic utilizing telemedicine services for initial evaluations, followed by in-person skin testing and/or oral challenge was implemented in a tertiary-care children’s hospital. Benchmark data of this piloted clinic was obtained via retrospective chart review of children evaluated between December 2021 and December 2022.

**Results:**

Following the implementation of a collaborative PCN allergy clinic utilizing telemedicine for initial evaluations, referral rates for pediatric PCN allergy evaluations increased from an average of 15 per month to 53 per month. Of 459 referrals, 355 (77.3%) were successfully scheduled in one of the telemedicine clinics, and 348/355 (98%) completed their initial telemedicine evaluation. 89 children who completed their recommended in-person formal evaluation were de-labeled, and 23 failed their challenge and remain labeled as penicillin allergic.

**Conclusion:**

Piloting a collaborative PCN allergy clinic within two ambulatory divisions of a tertiary care center and utilizing telemedicine services has resulted in a successful avenue to promptly evaluate and de-label children with PCN allergies. Future work will be needed to determine prescribing outcomes of children de-labeled in this PCN allergy clinic.

**Disclosures:**

**All Authors**: No reported disclosures

